# A gene variation of Interferon Gamma Receptor-I promoter (rs1327474A>G) and chronic hepatitis C virus infection 

**Published:** 2019

**Authors:** Maryam Karkhane, Seyed Reza Mohebbi, Afsaneh Sharifian, Amir Ghaemi, Hamid Asadzadeh Aghdaei, Mohammad Reza Zali

**Affiliations:** 1 *Basic and Molecular Epidemiology of Gastrointestinal Disorders Research Center, Research Institute for Gastroenterology and Liver Diseases, Shahid Beheshti University of Medical Sciences, Tehran, Iran*; 2 *Gastroenterology and Liver Diseases Research Center, Research Institute for Gastroenterology and Liver Diseases, Shahid Beheshti University of Medical Sciences, Tehran, Iran*; 3 *Foodborne and waterborne diseases research center, Research institute for gastroenterology and liver diseases, Shahid Beheshti University of Medical Sciences*; 4 *Department of Virology, Pasteur Institute of Iran, Tehran, Iran*

**Keywords:** Single nucleotide polymorphism, Chronic disease, HCV infection, IFNGR1

## Abstract

**Aim:**

In present survey, we attempted to inquire the plausible linkage of rs1327474 A/G and HCV chronic infection or the clearance of the virus.

**Background:**

IFN-γ signaling pathway is an important trigger for activating antiviral immune responses and production of wide variety of molecules with anti-microbial profiles including type 1 cytokines. Any defect or variation in IFNG signaling pathway may result in susceptibility or progression to diverse diseases such as inflammatory and virus associated disorders. Rs1327474 A/G also known as -611 A/G is an important variation which is located in promoter region of Interferon Gamma Receptor-I (IFNGR1) and may have potent risk for HCV susceptibility.

**Methods:**

For this purpose, 154 HCV patients and 200 controls were enrolled in the study, and genomic DNA was isolated from PBMCS and IFNGR1 -611 polymorphism was genotyped by polymerase chain reaction- fragments length polymorphism (PCR-RFLP) method.

**Results:**

While, AA, AG and GG genotypes frequency included 37.8%, 53.7%, 8.5% in healthy controls, 41.6%, 46.1%, 12.3% were found in chronic HCV patients. Interestingly, allelic percentage was similar in both separated groups within 64.7%, 35.3% and 65.3%, 34.7% were obtained for T and G allele in control and case group respectively.

**Conclusion:**

In spite of our exception for the possible role of this variation in an important promoter region of IFGR1 gene, rs1327474 A/G was not associated with HCV chronic infection among an Iranian studied group. Comprehensively, -611A/G cannot be considered as a risk biomarker for susceptibility to chronic HCV disease.

## Introduction

 Hepatitis C remains a major global public health concern. Incidence of HCV is approximately 170 million cases of world’s population (1). Despite low prevalence of HCV in Australia, Europe and American countries, yet African and the Eastern Mediterranean countries have reported higher incidence (2). Irrespective of region, most infected subjects improve chronic state and only approximately 20% of patients have a self-limited infection by a spontaneous viral RNA elimination during 6 months of infection (3). Thereupon, this finding indicates that certain individuals are more protected against HCV infection. Although numerous studies have been attempted to search about HCV infection and immune system interplay, exact mechanisms of HCV clearance have not been identified to date (4). Single nucleotide polymorphisms of several human genes were studied and proposed as association factors for chronic HCV infection susceptibility. So, genetic background including major cytokine and cytokine related genes variations may involve in HCV infection susceptibility within different ethnic groups (5, 6). 

Interferon gamma (IFN-ɣ) signaling has a pivotal role in both innate and adaptive immune responses against variety of stimuli included viral infections, other intracellular pathogens and tumors (7, 8). IFN-ɣ is markedly capable of inducing cellular resistance against viral attacks. This lymphokine is produced by T CD4+, T CD8+, NK, NKT and antigen presenting cells and functionally differs from type I interferon (9, 10). IFN-ɣ as a sole member of type II interferon profoundly triggers the activation of immune mediated inflammatory responses, causes to differentiation of naïve T cell to IFN-ɣ producing effector T cell and inhibition of Th2 proliferation, induces to Ig class switching, promotion of macrophage killing and up-regulation of MHC I & II (11). It has been proved that IFN-ɣ mediates an intracellular antiviral response in hepatitis B and C infection through induction of signaling cascade along with an autocrine manner. It caused the up-regulation of interferon regulatory factor 1 (IRF-1) and IFN stimulating genes and consequently, leading to amplification of IFN-ɣ dependent responses through a specific feedback loop. The above-mentioned properties of IFN-ɣ are accomplished via binding to IFNG receptor complex which consists of two chains of IFNGR1 and two chains of IFNGR2. Specific Janus kinases (Jak1 and Jak2) are activated after binding of IFN-ɣ to IFNGR complex, and phosphorylation and dimerization of STAT-1 occur. Activation of STAT-1 leads to its nuclear translocation and initiation of gene transcription, consequently (10). Collectively, IFN-ɣ suppresses the HCV replication through the following process within the activation of associated genes. Therefore, any variation in these related genes may affect the HCV clearance and/or progression to chronic disease. Previous studies on injecting drug users and acute HCV infection revealed the profound significance of IFN-ɣ in viral clearance. It seems that lower production of IFN-γ and IL-2 in early phases of HCV infection links to progression into chronic infection. On the other hand, HCV clearance is associated with a notably greater and immense specificity of IFN-γ response (12, 13). 

In addition, clinical importance of IFNGR pathway in immune responses has been addressed by several studies on IFNGR1 defects. Mutational variations in the IFNGR1 gene in patients or animal models may cause an immunodeficiency that can lead to inadequate response to intracellular infections such as viruses and bacteria (14).

However, aberrant production of IFN-ɣ is associated with auto inflammatory and autoimmune diseases (8). Also, IFNGR variations were investigated in Tuberculosis, Nontuberculous Mycobacterial Lung Infection, HBV, Listeria Monocytogenes, Toxoplasmosis infection, Autoimmune Arthritis, and cancers (15-22).

We tried to evaluate the IFGRI variation at -611 promoter position in chronic HCV patients and healthy individuals in order to study the potent role of IFNGRI gene polymorphism as a biomarker in HCV susceptibility and chronicity. Therefore, we expect to detect both of the HCV predisposing and protective alleles occurred in -611 within the promoter region of IFN-ɣR1, in Iranian population. 

## Methods

**Study population**

154 mono infected HCV patients according to inclusion criteria were studied as the case and 201 healthy individual’s blood donor considered as the control group. Patients had history for positive HCV Ab and detectable HCV viremia and also, were negative for HBsAg and HBcAb. The patients did not report any history for HIV and any other infectious diseases. Patients with consequence HCV-HBV and/or HCV-HIVco-infections were excluded from the study. 

In contrast to patient group, undetectable results for HBsAg, HCV Ab, HIVAb and HCV viremia were essential for control subjects which confirmed by ELISA (Dia.pro Diagnostic Bioprobes Kit, Italy) and nested following of RT-PCR (RevertAid RT kit, Thermo Scientific, USA), respectively. It is mentionable that ethical approval was obtained from every studied individual who participated in this research.

**Genotyping procedure of IFN-ɣ R1**

Genomic DNA was extracted from buffy coat in accordance to Sambrook’s instruction for DNA extraction by phenol-chloroform method (23). PCR experiment was performed as following condition; Initial denaturation 94 ˚C for 5 minutes, 35 cycles for 95 ˚C for 35 sec, 60˚C for 35 sec and 72 ˚C for 35 sec and finally, 72 ˚C for 10 minutes. Amplified partial IFNGR gene of all participants was subjected for genotyping which was based on RFLP method and implemented accordance to table 1. Amplicon with 260 bp in length were digested by Hpy1881 (New England Biolab) and genotype variability were visualized on agarose (peg Gold Universal Agarose, Belgium) based gel electrophoresis 3% which stained with ethidium bromide under UV light. 5% of samples were randomly sequenced (Macrogen Company, Korea) for validation of RFLP-genotyping set up.

**Table 1:** Details of genotyping by PCR-RFLP

**Polymorphism** **Position, chro6q23.3**	**Primer sequence (5’-3’)**	**Annealing temperature**	**Restriction** **enzyme**	**Allele** **phenotype**
-611 A>G	F:CTCTTCATGAGAGGCTGTCT R:TAACTCTTGGAGTTCACCTGG	60˚C	Hpy188I (5unit/reaction)	A: 260bp G:240 + 20bp

**Table 2:** Clinical and Demographic Data of Studied Population

	**Healthy individuals**	**HCV patients**	
Study population	201	154	
Male & female %	53.7% - 46.3%	78.6% - 21.4%	P<0.001
HBs Ag	Negative	Negative	
anti-HBc Ab	Negative	Negative	
Anti-HCV Ab	Negative	Positive	
ALT(U/L)	24.6±10.4	42.2+12.6	
AST (U/L)	22.8+9.6	40.4+14.1	
Mean Age ± SD (y)	44.95±1.16	46.02±1.21	P=0.19
Age range (Y)	17-85	16-81	

**Table 3:** Genotype distribution and allelic frequency of -611 gene polymorphisms in both of case and control groups

**SNP -611 G/A**	**Healthy subjects**	**HCV patients**	**Adi OR (CI:95%)**	**P-value**
TT	76 (37.8%)	64 (41.6%)	Reference	-
TC	108 (53.7%)	71 (46.1%)	0.67 (0.31-1.44)	0.31
CC	17 (8.5%)	19 (12.3%)	0.59 (0.28-1.26)	0.17
Allele				
T	260 (64.7%)	201 (65.3%)	Reference	-
C	142 (35.3%)	107 (34.7%)	1.03 (0.75-1.43)	0.83

**Statistical methods**

Data was analyzed by IBM SPSS statistics 21 and by the Chi-square, Mann-Whitney U non-parametric test, and Binary logistic regression examination. 95% confidence interval was assumed and under 0.05 levels for P-value was exactly considered as statistical significance criteria. 

## Results

The subjects under the study contained 201 healthy individuals as the control and 154 HCV patients who unequally distributed in the population based on the gender and age. So, male gender was significantly increased in the patients group (χ^2^<0.001, df:1, p<0.001). Analyzing of non-parametric data of age was seen without any statistical distinction. However, details of demographic information are described in table2.

The examination of gentyping results illustrated that not only -611 IFN-ɣR1 genotyping had considerable differences among two groups; interestingly, allelic frequency of case group did not alter in comparison with control group and both case and control groups had near allelic frequency. However, table 3 was addressed the genotype and allele distribution in case and control by separation.

## Discussion

Environmental factors contain HCV virulence, socioeconomic situation, drug/alcohol abuse, ethnicity and host genetic variation might elevate the risk of HCV infection (5, 24, 25). The most currently studies focused on INF type I and III in HCV susceptibility, and very little is known about the role of IFN type II(5, 26, 27). Whereas, our assay searched the potent acelator/protective role of -611 (rs1327474 A/G) IFN-ɣ R1 gene polymorphism in incidence of HCV infection. 

A series of studies were indicated IFNG and IFNGR1 variation in penetrance, susceptibility and severity of multiple inflammatory, virus and bacterial associated diseases (28).

Izad M *et al*. invistigeated the role of IFN-ɣ variation and severity of multiple scrosis in Iran, within found no statistical relation (29). Also, IFN-ɣR1 variation was not reported in association with risk and/or protective of pulmonary TB in Iranian population as well (30, 31). Nevertheles, in another report in chinease tibetan population, rs1327474 A/G have been found to be playing markedly role for susceptibility of TB (32). Khanizadeh S *et al.* studied IFN-ɣ R1 variation in Iranian HBV patients and implicated that beneficial linkage between lower risk of HBV and presence G allele in -611 posistion of IFNɣR1 (16).

Variation of IFNGR1 at -56 position was case reported by Velayati *et al.* in 2011 that caused hypersensivity to leprosy in children of single family in Iran (33). Furthermore, mice with IFNGR deficiency had higher risk for development of colorectal cancer and IFNGR might be considered as limiting factor against development of CRC (34). In accordance with contribution of IFNG in progression of inflammatory diseases, some studies addrressed this issue. In a study by Heidari *et al*., the correlation of rs1327474 and inflammatory periodontitis was investigated. However, no diffrence exsited in case and control groups of pervious study (35). To our knowledge, there is no study that spans around hepatitis C infection and IFNGR1 variation in Iranian population up to now. The outcomes of the present assay are consistent with other studies in the same population. So close results were observed for rs1327474 AA, AG, GG distrubtion and A /G allelic ferquency. Noteworthy, the results implicated the allelic frequency was based on hardiweinberg equilibrium in the population and both of alleles had equal precentage in this Iranian population.

Collectively, there were weak evidence for detectable linkage of -611 A/G IFNGR1 variation and HCV infection incidence for Iranian ethnicity. Although, IFNGR1 plays a key role in the IFN-γ signaling pathway, the studied polymorphism did not display an obvious correlation to HCV infection. Perhaps, more studies with greater sample size are necessary to confirm the protectivity or risk contribution of -611 A/G in HCV susceptibility in Iranian ethnicity. 
